# Prediction of low 5-minute Apgar scores: development and internal validation of parity-stratified clinical prediction models for sub-Saharan Africa

**DOI:** 10.1186/s12884-026-09153-7

**Published:** 2026-04-25

**Authors:** Michaela Franzén-Malmros, Shrouq Daraiseh, Stefanie Schmauder, Joseph Akuze, Phillip Wanduru, Bianca Kandeya, Kristi Sidney Annerstedt, Christian Agossou, Hussein Kidanto, Andrea Barnabas Pembe, Claudia Hanson, Sunjuri Sun

**Affiliations:** 1 Department of Global Public Health, Karolinska Institutet, Wideströmska Huset, Tomtebodavägen 18, Stockholm, 171 65 Sweden; 2 Clinical Epidemiology Division, Department of Medicine Solna, Karolinska Institutet, Stockholm, Sweden; 3 London School of Hygiene & Tropical Medicine, London, UK; 4 Center of Excellence for Maternal, Newborn and Child Health, Makerere University School of Public Health, Kampala, Uganda; 5 Kamuzu University of Health Sciences-Center for Reproductive Health, Blantyre, Malawi; 6 Centre de Recherche en Reproduction Humaine et en Démographie (CERRHUD), Cotonou, Benin; 7 Medical College East Africa, Aga Khan University, Dar es Salaam, Tanzania; 8 Muhimbili University of Health and Allied Sciences, Dar es Salaam, Tanzania

**Keywords:** Apgar score, Childbirth, Intrapartum care, Perinatal health, Predictive modelling methods, Sub-Saharan Africa

## Abstract

**Background:**

Low 5-minute Apgar scores remain an important indicator of compromised neonatal status and may assist in identifying high-risk newborns in resource-constrained or high-volume labour ward settings. Accurate prediction of newborns at risk could guide timely intrapartum and immediate postpartum interventions. Because risk factors vary by maternal parity, prediction models may benefit from a parity-specific approach. This study aimed to develop and internally validate two prognostic models for predicting low 5-minute Apgar scores, stratified by parity.

**Methods:**

The analysis used data from 124,376 singleton births at or beyond 28 weeks of gestation, recorded between July 2021 and December 2023 across 16 hospitals in Benin, Malawi, Tanzania and Uganda. Model predictors were selected using a knowledge-based approach, and multivariable logistic regression was performed. Model performance was assessed through calibration and discrimination with internal validation conducted using bootstrapping. The predicted outcome was the 5-minute Apgar score, categorised as low (< 7) or normal (≥ 7).

**Results:**

In the overall study population, 6.3% of newborns received a low Apgar score. The final nulliparous and parous models included 14 and 19 predictor parameters, respectively, with country included as an additional fixed effect. The models demonstrated moderate optimism-adjusted performance, with C-statistics of 0.663 for the nulliparous model (95% CI: 0.654**–**0.675) and 0.732 for the multiparous model (95% CI: 0.724**–**0.740). Calibration was excellent in both models, with calibration-in-the-large (CITL) values of 0.000–0.001 and calibration slopes of 0.989–0.995. Antepartum haemorrhage and severe anaemia were the strongest contributors in both models.

**Conclusions:**

Two prediction models for low 5-minute Apgar scores, one for nulliparous and one for parous women, demonstrated moderate predictive ability. External validation and further testing are necessary to assess the generalisability and clinical utility of these models.

**Supplementary Information:**

The online version contains supplementary material available at 10.1186/s12884-026-09153-7.

## Background

In sub-Saharan Africa, the burden of neonatal morbidity is significantly higher and the neonatal mortality rate is more than ten times higher than in high-income countries [1]. Systemic barriers, such as inadequate neonatal facilities and poor health infrastructure create bottlenecks that contribute to a disproportionate burden of neonatal complications in this region [2]. Intrapartum-related events, including birth asphyxia, account for approximately 23% of all neonatal deaths. Early identification of newborns at risk is essential, as birth complications are associated with both increased neonatal mortality and severe long-term disabilities [3].

The Apgar score, developed in the 1950s [4] is widely recognised as the global standard for the immediate assessment of newborns [5, 6]. Risk factors such as low birth weight [7–9] and prolonged labour [10] have been associated with a low 5-minute Apgar score. A low 5-minute Apgar score at birth may be associated with increased likelihood of neonatal mortality [11], particularly amongst preterm infants [12],as well as long-term neurological disability and impaired cognitive function [13]. While studies from high-income countries often subdivide low Apgar scores into moderate and severe categories, this distinction may be less relevant in low-resource settings in sub-Saharan Africa, where early neonatal mortality is high and may reduce prognostic differences between categories. Mothers have different risk profiles depending on their parity status, which is known to influence clinical decision-making and baseline risk assessment. Parous mothers (those who have given birth before) have a different risk profile compared to nulliparous mothers (those giving birth for the first time) when it comes to adverse birth outcomes [14].

Risk assessment at admission for childbirth and consequent decisions on the type and intensity of follow-up and care has a long tradition in obstetrics [15]. However, the intrapartum care guidelines by the World Health Organization are silent on risk criteria [16] and few projects in sub-Saharan Africa have investigated criteria to decide on the type and intensity of care at admission to hospitals [17]. Prediction modelling to inform clinical decision has gained large attention globally [18], resulting in a rapid development of obstetric prediction models. These models are most often developed for high-income countries [9, 19] and depend on predictors that are not as readily available, such as those measured by obstetric ultrasound [9, 20, 21]. This represents a missed opportunity, as the identification of newborns at risk is particularly critical in the context of resource-limited or high-demand labour wards. In low-resource settings, admission represents a critical decision point at which limited resources, such as skilled birth assistants, monitoring capacity and neonatal resuscitation equipment must be allocated according to clinical need rather than availability or routine practice. Women identified as being at high risk of delivering a newborn with a low Apgar score could therefore be prioritised for closer assessment, enhanced monitoring, and delivery near neonatal resuscitation capacity. In contrast, women classified as low risk could be safely managed with standard monitoring protocols, thereby supporting more efficient and equitable use of constrained resources.

### Study objective

Predicting low 5-minute Apgar scores using labour admission data could improve patient outcomes by increasing the possibility of efficient resource allocation in under-resourced delivery rooms in sub-Saharan Africa. To date, no prediction model has utilised data from a large multinational registry from sub-Saharan Africa to predict low 5-minute Apgar scores. Furthermore, no such model has been stratified by parity status in this setting. The intended clinical use of the model is to support decision-making at the time of admission for hospital-based childbirth in a resource-constrained setting. By providing a standardised risk assessment, the model could complement clinical judgement and enable consistent and timely implementation of the most appropriate clinical approach.

The aim of this study was to develop and internally validate two models for predicting low 5-minute Apgar scores, one for nulliparous and one for parous women, using labour admission data collected from 16 hospitals in Benin, Malawi, Tanzania and Uganda. By stratifying models according to parity, the study further aims to enhance clinical applicability, recognising that risk profiles differ substantially between nulliparous and parous women. This study focuses on prediction rather than causal inference; thus, the included variables should not be interpreted as having causal effects on the outcome.

## Methods

### Study design and setting

This prognostic predictive modelling study was based on data collected as part of the Action Leveraging Evidence to Reduce Perinatal Mortality and Morbidity in Sub-Saharan Africa (ALERT) project, which was a cluster randomised stepped-wedge trial and included data collection for a clinical perinatal e-registry. The registry data were originally collected for clinical and research purposes rather than specifically for prediction modelling. Sixteen hospitals from Benin, Malawi, Tanzania and Uganda were included based on the criteria that they report more than 2500 births per year and offer caesarean section and blood transfusion services [22]. Eligible hospitals were either public or private not-for-profit and predominantly located in rural areas, except for one included tertiary-level university hospital [23]. Once eligible study participants were identified, data were prospectively entered into the ALERT perinatal e-registry by hospital staff (doctors, nurses, midwives or data clerks) abstracting data from antenatal cards, admission books, birth records or notes and postnatal registers. Data controllers at each hospital performed daily data checks and verifications, which were reviewed weekly by the national data controllers and the ALERT data controller at Karolinska Institutet throughout the data collection period. Patients and the public were not involved in the design, conduct or dissemination of this prediction model study but involved into the ALERT study. A protocol of the ALERT study with detailed information on the perinatal e-registry has already been published [22, 24].

### Study population

Data on births that took place between 1 July 2021 and 31 December 2023 in 16 hospitals in Benin, Malawi, Tanzania and Uganda were entered into the ALERT perinatal e-registry. Pregnant women at ≥ 28 weeks of gestation and with newborns weighing more than 1000 g at birth were included for data collection. In line with the aim to develop a model defining risk of adverse events at admission to childbirth, women who experienced an induced labour due to foetal death confirmed by a negative foetal heartbeat on admission were excluded, as the negative event was already known. Multiple pregnancies were also excluded because of the exclusive risk profile. Observations with missing values for gestational age, parity and the outcome variable (5-minute Apgar score) were also excluded from the analysis.

In this setting, negative foetal heartbeat at admission is not considered a reliable clinical indicator of foetal viability, as routine ultrasound examinations and other appropriate monitoring methods are not available to confirm [25]. Consequently, presence or absence of a detectable foetal heartbeat at admission does not systematically guide clinical management in this setting. In the ALERT perinatal e-registry, foetal heartbeat at admission is recorded as either positive, negative or not documented [22]. However, this variable does not determine or influence subsequent management decisions in routine practice. Excluding cases based on foetal heartbeat status at admission could therefore introduce selection bias without reflecting actual differences in clinical care pathways.

### Outcome

The predicted outcome was the 5-minute Apgar score [4] assigned to a newborn five minutes after birth. The Apgar score is included in several core outcome sets, such as those for labour and delivery management [26] and labour induction [27]. It is also included in a core outcome set as part of the definition of birth asphyxia [28]. It is recognised as important by key stakeholders, such as pregnant women and clinicians, and its relevance in low-resource settings has also been highlighted [27]. During data collection, the score was recorded by hospital staff in the perinatal e-registry as an ordinal variable ranging between 0 and 10. Data for this variable were missing for 0.3% of births within the data collection period. For predictive modelling, the recorded value was dichotomised into the categories low (< 7) and normal (≥ 7). This is consistent with common practice in clinical research, as an Apgar score below 7 signals the threshold at which concern is warranted [29, 30].

### Candidate predictors

A knowledge-driven approach was used to select eligible predictors based on clinical reasoning, existing evidence [9, 10, 19, 20] and the availability of variables in the dataset. Predictors were selected based on their availability at the time of admission, as the aim of the model was to enable risk prediction at this stage (i.e., before the onset of labour and prior to the occurrence of intrapartum factors). Therefore, all predictors included in the final model were available at admission, in accordance with the purpose of the prediction model. For the purpose of this study, data was continuously entered into the ALERT perinatal e-registry by data clerks or midwifes in maternity wards, including indicators collected during the antenatal period. A more detailed summary including the methods and timing of measurement has already been published [22].

Predictor inclusion was guided by predictive utility in accordance with current methodological recommendations for prediction modelling [31]. In prediction models, predictors are included based on their contribution to overall predictive performance rather than on statistical significance. In this study, predictor selection was performed using the Least Absolute Shrinkage and Selection Operator (LASSO). The penalty parameter (λ) was determined using 10-fold cross-validation. A sequence of 100 candidate λ values was evaluated, and the optimal λ was chosen as the value minimising the cross-validated prediction error. LASSO was used solely for variable selection, and coefficient estimates in the final model were obtained without penalisation. Some predictors retained by LASSO and contributing to overall model performance may exhibit confidence intervals that include zero. Selecting predictors based on significance testing may introduce selection bias and increase the risk of model overfitting, ultimately harming model performance.

A final list of selected model predictors can be found in Supplementary Table 1. The selected predictor variables included: antenatal care visits, antenatal haemorrhage, cardiac/renal disease, diabetes mellitus or gestational diabetes, gestational age, human immunodeficiency virus (HIV), hypertensive disorders, last pregnancy outcome, malaria, maternal age, parity, pre-labour membrane rupture, previous caesarean section, severe anaemia and syphilis. The categorical variables were coded with the corresponding reference categories, i.e. last pregnancy outcome (baby alive at 28 days as reference), onset of labour (spontaneous as reference), HIV (negative as reference) and syphilis (negative as reference). For the HIV and syphilis variables, the option “not recorded” was listed as a separate category. All other variables were binary with the options yes or no (no as reference).

### Analysis

All data analyses were performed using Stata/BE18.8 (StataCorpLLC, College Station, TX, USA). This study followed the guidelines for Transparent Reporting of a multivariable prediction model for Individual Prognosis Or Diagnosis (TRIPOD) [31], with the corresponding checklist available in the Supplementary Table 2. Multivariable logistic regression models with country fixed effects were used to control for unobserved, time-invariant differences in recorded 5-minute Apgar scores between countries. Separate models were developed for nulliparous and multiparous women given differences in baseline risk and labour physiology. Logistic regression remains the recommended approach for clinical prediction models due to better interpretability, relevance and implementation in practice [32], and several studies have demonstrated similar or improved performance as compared to machine learning algorithms [33, 34]. For this study, models including random effects for hospitals were also evaluated; however, as this introduced increased model complexity without improving predictive performance, they were not retained.

As the proportion of missing data for all predictor parameters was low (< 1%), a complete case analysis was performed. No collinearity was found between any of the predictors, allowing for the use of an additive model. Possible non-linear relationships were examined using simple transformations (quadratic or cubic) for the continuous variables for maternal age and gestational age. However, this did not lead to an improvement in model fit, so these variables were retained without transformation. The appropriate sample size was determined using the *pmsampsize* Stata package, following the methodology described by Riley et al. [35]. Calculations were performed based on the development of a model for a binary outcome with an expected C-statistic of 0.78, derived from a previous study [20]. The prevalence of the outcome was 6.3% and a maximum of 25 predictor parameters were considered. Based on this, the minimum sample size was calculated to be 3279 participants with 207 events (occurrence of a low 5-minute Apgar score). The final study population exceeded the minimum sample size required for this approach.

The predictive performance of each model was evaluated using calibration and discrimination measures. Model discrimination was assessed by calculating the area under the receiver operating characteristic (AUROC) curve, which corresponds to the concordance statistic (C-statistic) for logistic prediction models [36]. The model calibration was assessed using the ratio of expected-to-observed (E:O) events, calibration-in-the-large (CITL) and the calibration slope. CITL is the intercept of a fitted logistic regression model that compares the averages of the predicted risk and the observed risk and equals 0 if there is no calibration bias (over- or underestimation of risk). The calibration slope measures the risk calibration over the entire range of predictions and indicates whether the predictions are too extreme (overfitting) or too conservative (underfitting). A calibration slope of 1 indicates a perfect calibration, while 0 is a flat line indicating that the model has no predictive power. In addition, the overall performance of the models was assessed using the scaled Brier score percentage and Nagelkerke’s R². For internal validation, bootstrapping was performed to assess the apparent and optimism-adjusted performance of the predictive models, using the *bsvalidation* package in Stata [37]. To assess the potential instability of the model, 500 bootstrap samples were drawn from the original dataset. Predictions were compared with those of the original model, and optimism was estimated as the difference between these two and averaged across all repetitions. The performance metrics were then corrected for optimism. The overall workflow for model development and validation is illustrated in Supplementary Fig. 1. The logistic regression equation defined as *P* = 1/ (1 + exp (-(Z)), where Z = β0 + β1 × 1 + β2 × 2+⋯+βpXp, was used to calculate the predicted probability of a low 5-minute Apgar score. The full model specification is provided in Supplementary Tables 3–4.

Additionally, we performed decision curve analysis to evaluate the clinical utility of the models across a range of threshold probabilities. Nomograms were developed to facilitate the practical implementation of the prediction models. A sensitivity analysis was performed, excluding pregnancies with a documented negative foetal heartbeat on admission. A second sensitivity analysis was conducted to assess the potential impact of the ALERT intervention on model performance by comparing the intervention and control groups. A third sensitivity analysis evaluated the potential added utility of ultrasound in this setting, incorporating ultrasound-derived predictors by fitting an expanded model including fetal presentation (breech or transverse) and birthweight. Finally, model transportability was assessed for the parous model using internal–external cross-validation, iteratively leaving one country out for validation.

## Results

**Fig. 1:**
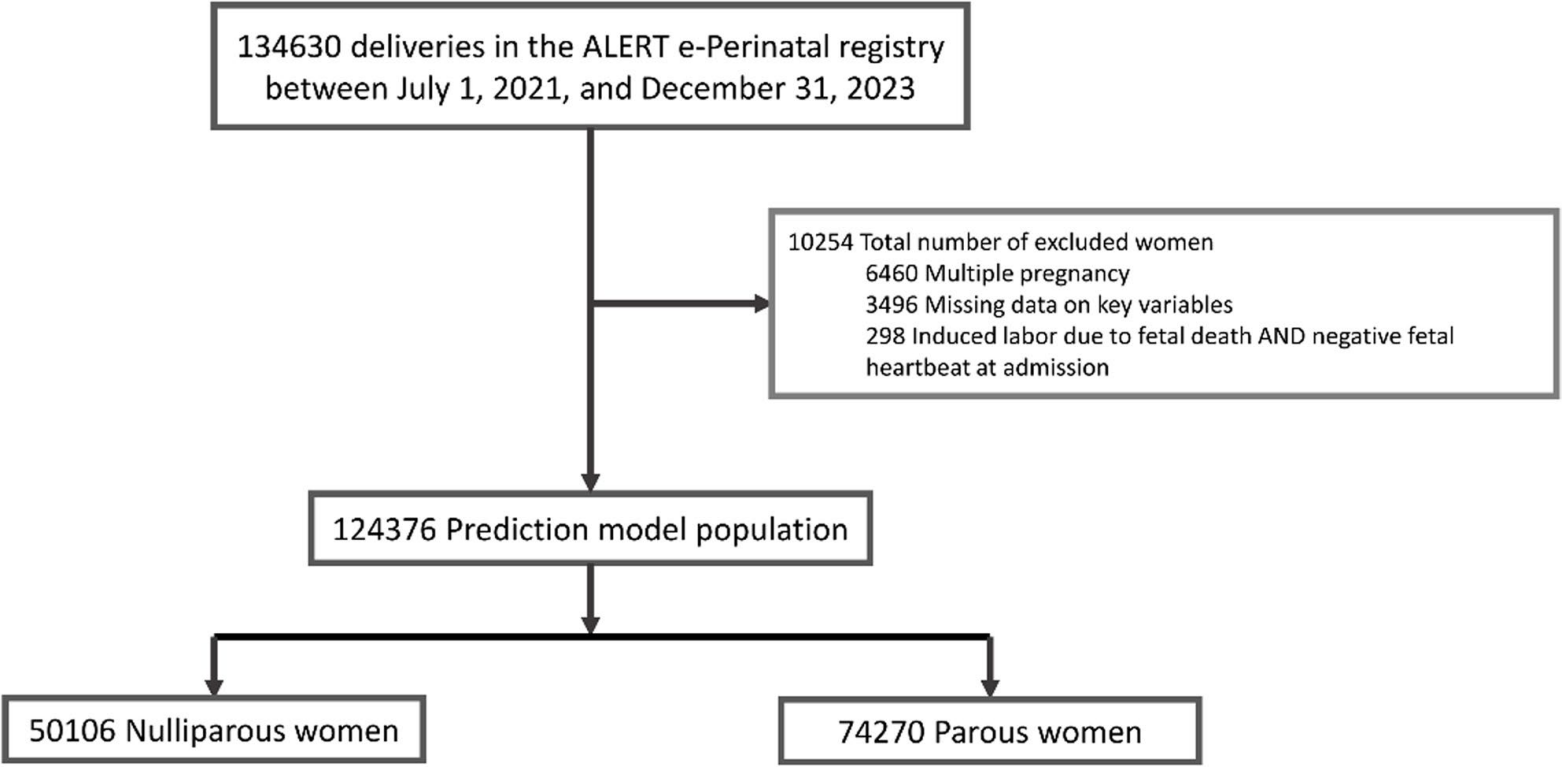
Flowchart for the inclusion of participants in parity-stratified prediction models

The selection of the final study population for the parity-stratified prediction models is described in Fig. 1. Of 134,630 births, 124,376 singleton births were included for prediction modelling, including 50,106 (40.3%) nulliparous women and 74,270 (59.7%) parous women. The age distribution of participating women ranged from 13 to 50 years, with a younger median age for nulliparous women (19 years, IQR 18–22) compared to parous women (28 years, IQR 24–32). Women in both groups attended a median of four antenatal visits for the current pregnancy and were a median of 38 weeks pregnant when they were admitted. The referral rate was higher for nulliparous women (26.7%) than for parous women (22.7%). Of the women who were parous, 20.8% had experienced a caesarean section in the past. Regarding the outcome of their last birth, 7.2% had a miscarriage or abortion, 1.5% had a stillbirth, 1.3% had a neonatal death and 89.1% had a baby that was still alive after 28 days. Across the entire study population, 6.3% of newborns received low Apgar scores. Among these, 30.6% were fresh stillbirths and 21.5% were macerated stillbirths. Of the nulliparous women, 6.1% had a 5-minute Apgar score below 7, compared to 6.4% for the parous women. A summary of the baseline characteristics of the study population stratified by parity can be found in Table 1. A comparison of country- and hospital-specific population characteristics can be found in Supplementary Table 5.

**Table 1:** Summary of parity-stratified maternal and clinical characteristics among mothers giving birth in 16 hospitals in Benin, Malawi, Tanzania and Uganda between June 2021 and December 2023 (*n* = 124376)

Characteristics	Nulliparous		Parous		Missing data
	*n* or median	% or IQR	*n* or median	% or IQR	%
	50,106	40.3	74,270	59.7	
Current pregnancy and delivery					
Maternal age (years)	19	(18–22)	28	(24–32)	0.1
Gestational age (weeks)	38	(37–39)	38	(37–40)	0.0
Antenatal care visits	4	(3–5)	4	(3–5)	0.5
Parity (including stillbirths)	NA**	NA	2	1–3	0.0
Referred*	13,373	26.7	16,860	22.7	0.2
Antepartum haemorrhage	256	0.5	949	1.3	0.0
Pre-labour membrane rupture	1063	2.1	1836	2.5	0.0
Low 5-minute Apgar score (< 7)	3053	6.1	4757	6.4	0.0
Maternal health conditions					
Hypertensive disorders	2861	5.7	3884	5.2	0.0
Cardiac/renal diseases	68	0.1	143	0.2	0.0
Diabetes mellitus	92	0.2	193	0.3	0.0
Gestational diabetes	76	0.2	133	0.2	0.0
Syphilis	193	0.4	433	0.6	0.0
HIV	679	1.4	2842	3.8	0.0
Malaria	587	1.2	639	0.9	0.2
Severe anaemia	287	0.6	537	0.7	0.1
Previous births					
Previous caesarean section	NA	NA	15,444	20.8	0.0
Last pregnancy outcome					0.6
Abortion/miscarriage	3559	7.1	5312	7.2	
Stillbirth	NA	NA	1101	1.5	
Neonatal death	NA	NA	963	1.3	
Baby alive at 28 days	NA	NA	66,141	89.1	

*Referred = transferred from another facility or provider (not self-presenting). **NA = Not Applicable

**Fig. 2:**
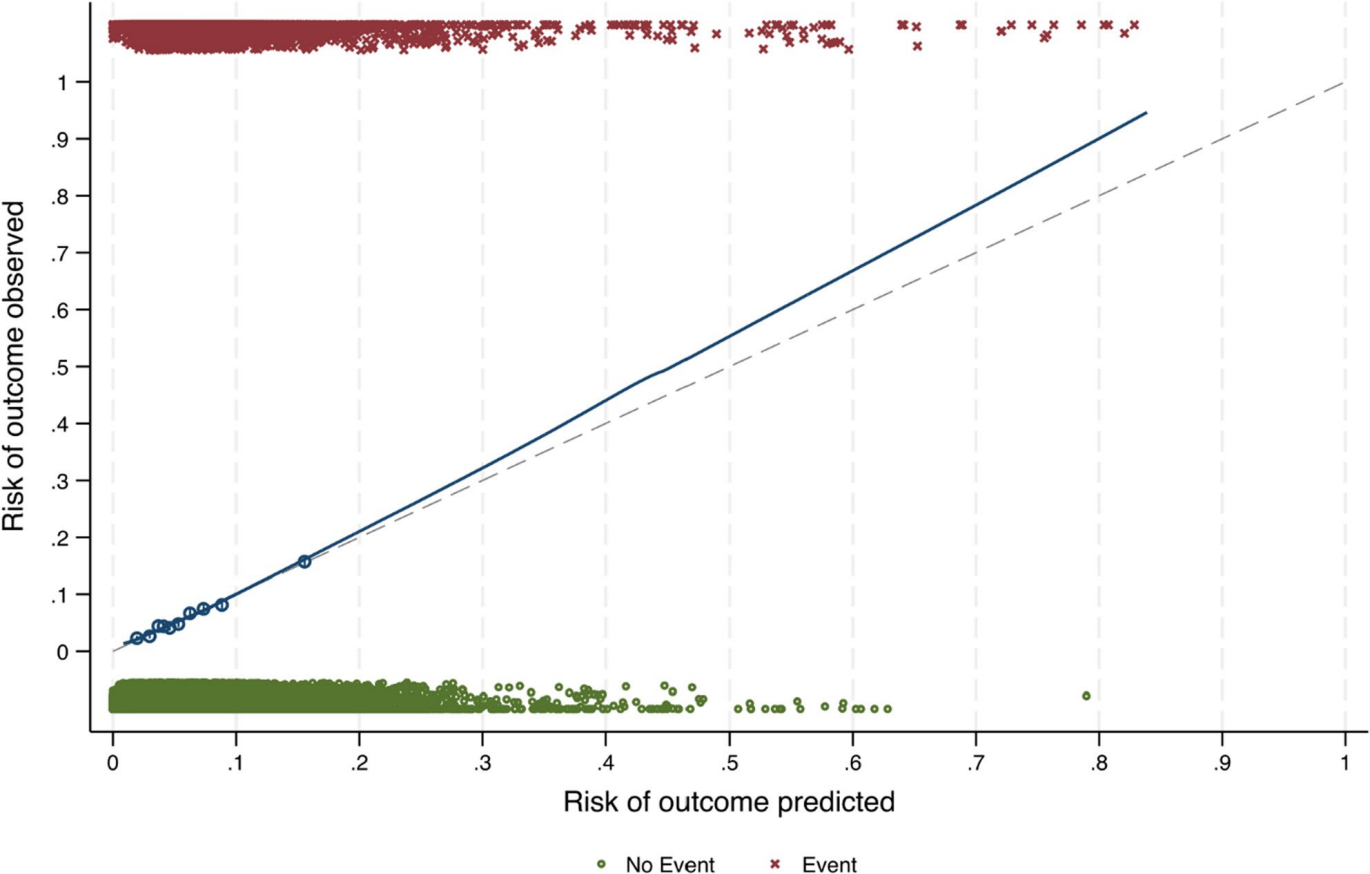
Optimism-adjusted calibration plot for prediction model for low 5-minute Apgar scores in nulliparous mothers (*n* = 50106)

The prediction model for nulliparous women had a C-statistic of 0.665 (95% CI: 0.654**–**0.675), indicating fair discriminatory power. LASSO penalisation led to the exclusion of the miscarriage/abortion parameter, indicating limited additional predictive value beyond the other included predictors. The final model included 14 predictor parameters, with country additionally included as a fixed effect; these are presented in Table 2 with their corresponding coefficients and risk probability formulae. The reported coefficients are intended for prediction purposes and should not be interpreted as estimates of causal effects. The E:O ratio was 1, indicating a balanced distribution of events and non-events. The CITL score of 0.000 (95% CI: -0.038**–**0.038) and the calibration slope of 1.000 (95% CI: 0.946**–**1.054) indicated excellent calibration. The scaled Brier score was 4%, indicating a relatively low prediction error. Nagelkerke’s R² was 0.05, indicating weak to moderate explanatory power. Optimism-adjusted metrics included a C-statistic of 0.663 (95% CI: 0.654**–**0.675), a scaled Brier score of 3.9%, an E:O ratio of 0.993 (95% CI: 0.959–1.033), the CITL at 0.001 (95% CI: -0.040**–**0.039) and the calibration slope 0.989 (95% CI: 0.941**–**1.045). The optimism-adjusted calibration plot for the nulliparous model is shown in Fig. 2. Minor deviation at higher predicted risks indicates a slight overestimation in these areas, but the model shows a reliable risk assessment overall.

**Table 2:** Model predictor coefficients before and after internal validation. A total of 14 predictor parameters were included in the nulliparous model and 19 in the parous model, with country included as an additional fixed effect. The table presents both the original regression coefficients from the fitted model and the optimism-adjusted coefficients obtained after bootstrap-based shrinkage; the latter represent the final coefficients corrected for overfitting

Predictors			Nulliparous model					Parous model				
			Coefficient (β)	Odds ratio	Optimism-adjusted Coefficient* (β)	(95% Confidence Interval)		Coefficient (β)	Odds ratio	Optimism-adjusted Coefficient** (β)	(95% Confidence Interval)	
Maternal age			−0.014	0.986	−0.014	(− 0.025,	−0.003)	−0.010	0.990	−0.010	(− 0.017,	−0.003)
Gestational age			−0.128	0.880	−0.127	(− 0.142,	−0.111)	−0.195	0.822	−0.195	(− 0.207,	−0.182)
Antenatal care visits			−0.050	0.951	−0.049	(− 0.072,	−0.026)	−0.116	0.890	−0.115	(− 0.135,	−0.096)
Parity			NA	NA	NA	NA	NA	0.121	1.128	0.120	(0.096,	0.144)
Antepartum haemorrhage			1.704	5.494	1.685	(1.409,	1.961)	1.991	7.326	1.981	(1.830,	2.133)
Pre-labour membrane rupture			−0.085	0.918	−0.084	(− 0.298,	0.129)	−0.155	0.857	−0.154	(− 0.314,	0.006)
Hypertensive disorders			0.304	1.355	0.301	(0.171,	0.431)	0.434	1.543	0.431	(0.324,	0.538)
Cardiac/renal diseases			−0.055	0.946	−0.054	(− 0.879,	0.770)	0.037	1.037	0.036	(− 0.484,	0.557)
Diabetes mellitus or gestational diabetes			−0.709	0.492	−0.701	(− 1.382,	−0.020)	0.002	1.002	0.002	(− 0.386,	0.390)
Syphilis Reference: Negative												
Positive			0.521	1.684	0.516	(− 0.016,	1.048)	0.576	1.779	0.573	(0.191,	0.955)
Unknown			0.223	1.250	0.221	(0.134,	0.307)	0.141	1.151	0.140	(0.066,	0.214)
HIV Reference: Negative												
Positive			−0.137	0.872	−0.136	(− 0.490,	0.219)	−0.278	0.757	−0.277	(− 0.470,	−0.084)
Unknown			0.206	1.229	0.204	(0.081,	0.326)	0.238	1.269	0.237	(0.133,	0.341)
Malaria			0.283	1.328	0.280	(0.006,	0.555)	0.050	1.051	0.050	(− 0.250,	0.349)
Severe anaemia			0.858	2.358	0.848	(0.545,	1.152)	0.907	2.477	0.902	(0.675,	1.129)
Previous caesarean section			NA	NA	NA	NA	NA	−0.444	0.641	−0.442	(− 0.525,	−0.359)
Last pregnancy outcome Reference: Baby alive at 28 days												
Abortion/miscarriage			NA	NA	NA	NA	NA	0.147	1.156	0.146	(0.033,	0.260)
Stillbirth			NA	NA	NA	NA	NA	0.505	1.657	0.502	(0.314,	0.691)
Neonatal death			NA	NA	NA	NA	NA	0.277	1.319	0.276	(0.043,	0.508)
Country Reference: Tanzania												
Benin			1.292	3.642	1.278	(1.112,	1.444)	1.283	3.607	1.276	(1.144,	1.408)
Malawi			0.356	1.428	0.352	(0.192,	0.512)	0.128	1.137	0.127	(− 0.011,	0.266)
Uganda			0.979	2.662	0.968	(0.805,	1.132)	0.809	2.246	0.805	(0.670,	0.941)

*Multiplied by the shrinkage factor = 0.987. Model intercept (β_0_) = 1.659. **Multiplied by the shrinkage factor = 0.995. Model intercept (β_0_) = 4.305

The formula to calculate the risk of a low 5-minute Apgar score is 1/(1 + e^-Z). Variables are coded 0 or 1 for absent or present respectively, except for the continuous variables: maternal age, antenatal care visits, parity and gestational age

Formula for nulliparous women: Z = 1.659 − 0.014 * maternal age + 0.513 * syphilis positive + 0.219 * syphilis unknown − 0.135 * HIV positive + 0.202 * HIV unknown + 0.279 * malaria + 0.299 * hypertensive disorders − 0.054 * cardiac/renal diseases − 0.697 * diabetes mellitus or gestational diabetes + 0.843 * severe anaemia − 0.049 * antenatal care visits − 0.126 * gestational age − 0.084 * pre-labour membrane rupture + 1.675 * antepartum haemorrhage + 1.270 * Benin + 0.350 * Malawi + 0.962 * Uganda

Formula for parous women: Z = 4.305 − 0.010 * maternal age + 0.120 * parity + 0.572* syphilis + 0.140 * syphilis unknown − 0.277 * HIV positive + 0.237 * HIV unknown + 0.050 * malaria + 0.431 * hypertensive disorders + 0.036 * cardiac/renal diseases + 0.002 * diabetes mellitus or gestational diabetes + 0.901 * severe anaemia + 0.146 * abortion/miscarriage + 0.502 * stillbirth + 0.275 * neonatal death − 0.442 * previous C-section − 0.115 * antenatal care visits − 0.194 * gestational age − 0.154 * pre-labour membrane rupture + 1.979 * antepartum haemorrhage + 1.275 * Benin + 0.127 * Malawi + 0.804 * Uganda

**Fig. 3:**
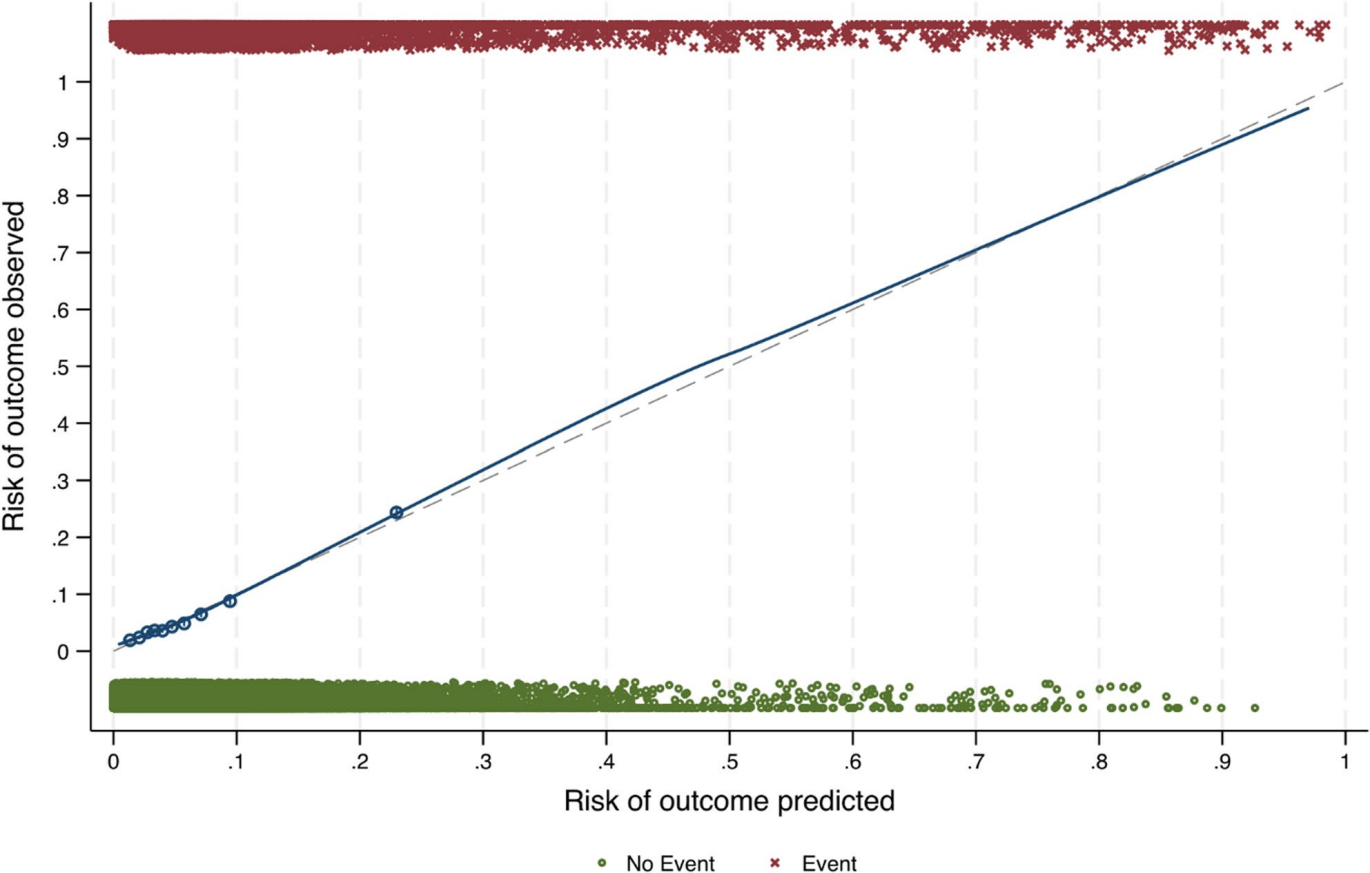
Optimism-adjusted calibration plot for prediction model for low 5-minute Apgar scores in parous mothers (*n* = 74270)

The prediction model for parous women had a C-statistic of 0.733 (95% CI: 0.725**–**0.741**)**, indicating good discriminatory ability. LASSO penalisation retained all candidate variables, suggesting that each predictor contributed independent predictive value to the model. The final model included 19 predictor parameters, with country additionally included as a fixed effect; these are presented in Table 2 with their corresponding coefficients and risk probability formulae. The E:O ratio was 1, indicating a balanced distribution of events and non-events. The CITL score of 0.000 (95% CI: **-**0.031**–**0.031) and the calibration slope of 1.000 (95% CI: 0.969**–**1.031) indicate excellent calibration. The scaled Brier score was 10.7%, indicating a relatively low prediction error. Nagelkerke’s R² was 0.14 indicating weak to moderate explanatory power. The optimism-adjusted metrics were a C-statistic of 0.732 (95% CI: 0.724**–**0.740), a scaled Brier score of 10.6%, an E:O ratio of 0.993 (95% CI: 0.969–1.016) the CITL at 0.000 (95% CI: **-**0.032**–**0.028) and the calibration slope 0.995 (95% CI: 0.962**–**1.028**).** The optimism-adjusted calibration plot for the parous model is shown in Fig. 3. The plot indicates good agreement between predicted and observed probabilities across most risk levels, with the calibration curve closely following the 45-degree line of perfect calibration.

**Fig. 4:**
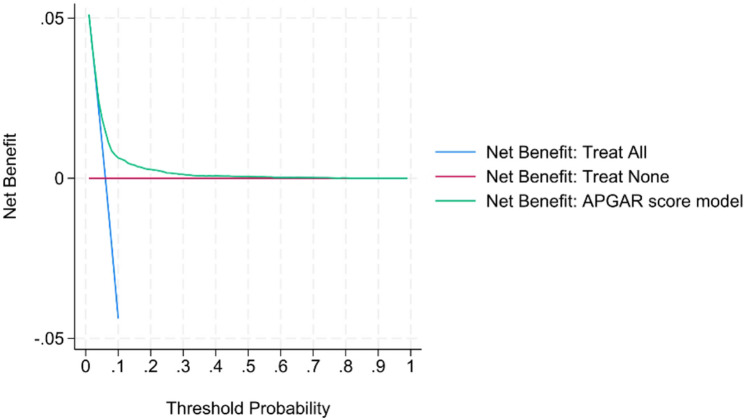
Decision curve analysis for nulliparous model

**Fig. 5:**
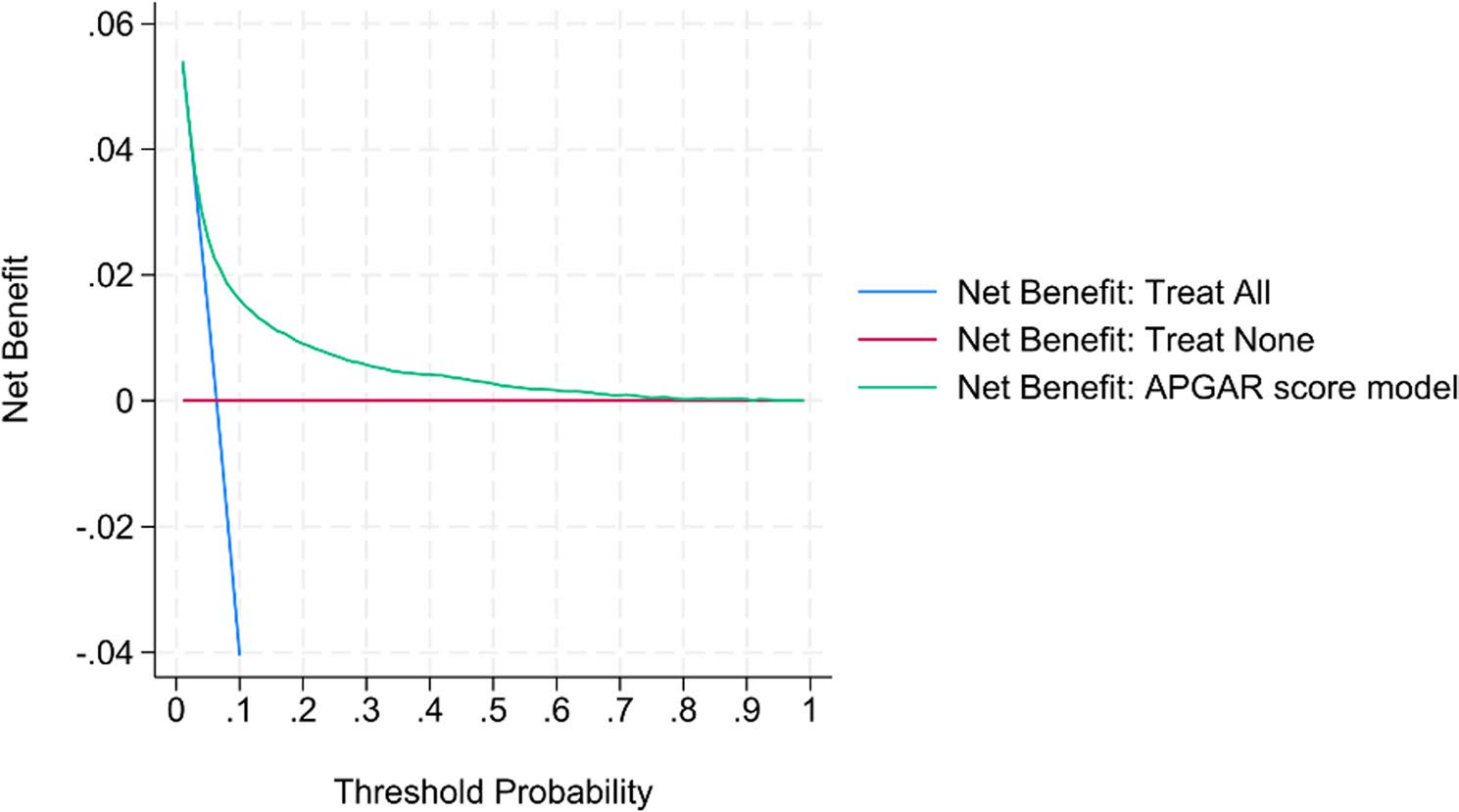
Decision curve analysis for parous model

The results of the decision curve analysis, shown in Figs. 4 and 5, suggest that the APGAR score model provides a higher net benefit than both “treat all” and “treat none” strategies for both nulliparous and parous groups if the preferred threshold probability is between approximately 0.05 and 0.3. This would encompass clinically relevant decision thresholds for the outcome of low Apgar score, as the positive prediction of an at-risk infant and subsequently enacting closer monitoring carries a low risk compared to the consequences of potentially missing an at-risk infant. To facilitate clinical implementation, individual risk can be estimated using the nomograms in Supplementary Figs. 2 and 3.

Overall, 1.6% of study participants had missing data for at least one predictor. We examined whether missingness was associated with any other variable using a logistic regression model with the “any missing” indicator as the outcome. As shown in Supplementary Table 6, no statistically significant associations were observed, and estimates were imprecise due to the low proportion of missing data, suggesting that any bias introduced by missingness is unlikely to be substantial.

Exclusion of pregnancies with a documented negative foetal heartbeat on admission did not improve predictive performance of models, as shown in Table 3. In the nulliparous model, 1,191 participants were excluded, including 400 with a live birth outcome. In the parous model, 2,437 participants were excluded, including 566 with a live birth outcome.

**Table 3:** Comparison of model discrimination (AUROC) with and without exclusion of pregnancies with negative foetal heartbeat on admission

	All pregnancies	Excluding negative foetal heartbeat on admission
Nulliparous model	0.662	0.645
Parous model	0.732	0.692

Both models included the *country* variable as a fixed effect to account for country-level differences. A comparison of model discrimination between country fixed-effects models and hospital random-effects models is presented in Supplementary Table 7. The optimism-adjusted coefficients were only slightly weaker compared to the coefficients before optimism-adjustment, indicating a minimal overfitting of the model to the development data. Model performance was similar between the ALERT control and intervention groups, with a maximum absolute difference in C-statistic of 0.021, indicating no meaningful effect of the intervention on predictive performance (Supplementary Table 8). A sensitivity analysis incorporating the two additional predictors foetal presentation and birth weight led to modest improvements in both models, with C-statistics increasing from 0.663 to 0.675 and from 0.733 to 0.754. Corresponding results are shown in Supplementary Figs. 4 and 5 and Supplementary Tables 9 and 10. Internal–external cross-validation indicated similar discrimination across countries, except the lower performance observed in Malawi, and calibration slope demonstrated modest between-country variation (Supplementary Table 11). However, substantial between-country heterogeneity was observed in calibration-in-the-large, suggesting differences in baseline risk across settings.

## Discussion

### Main findings

Using labour admission data from 124,376 women, two parity-stratified prediction models were developed. The final models included fourteen predictors in the nulliparous model and nineteen in the parous model, with country included as an additional fixed effect. Antenatal haemorrhage and severe anaemia emerged as the strongest predictors of low 5-minute Apgar score in both parity-specific models. After internal validation, the model for parous women showed better optimism-adjusted discrimination (AUC = 0.732) and higher overall accuracy (scaled Brier score = 10.6%) compared to the model for nulliparous women (AUC = 0.663; scaled Brier score = 3.9%). Although both models showed promise, especially in terms of calibration, the model for parous women performed better overall. The difference in scaled Brier scores indicates that the parous model explains a substantially greater proportion of outcome variability and has better overall predictive accuracy than the nulliparous model. This is likely due to the availability of more relevant anamnestic factors, such as information related to previous births [38]. Although the discrimination of the nulliparous model was modest, its clinical utility and impact remain uncertain and should be further evaluated in the context of external validation and further implementation science trials before conclusions about its applicability in practice can be drawn. The results support the hypothesis that it is possible to predict a low 5-minute Apgar score based on readily available pre-birth admission data. This indicates the potential benefit of such a prediction model for the early identification of at-risk women in low-resource settings.

### Strengths and limitations

A large, multinational perinatal registry from four countries in sub-Saharan Africa was used for this study. Data were collected from hospitals of different sizes and levels of care, selected to represent the diversity of the region, which improves the generalisability of the models. Data quality was promoted by having controllers at each hospital performing daily data checks and verifications, which were reviewed weekly by the national data controllers and the ALERT data controller at Karolinska Institutet throughout the data collection period. The robustness of the models was further supported by a sensitivity analysis, which found no clear evidence of a significant effect of the ALERT intervention on predictive performance.

To our knowledge, these are the only prediction models developed for low 5-minute Apgar scores using labour admission data from a large multinational registry of women in sub-Saharan Africa. In a systematic review comparing 21 prognostic models for adverse birth outcomes in LMICs, only one study included Apgar score as a predictor [39] and none as an outcome [40]. Existing models for predicting the Apgar score have been developed for high-income countries [9, 19] or depend on predictors that are not as readily available, such as gestational age or foetal heart rate measured by obstetric ultrasound [9, 20, 21]. A predictive model for low 5-minute Apgar scores after induction of labour in vaginal deliveries was previously developed for a low-resource setting in Tanzania. In this case the variables birth weight, maternal age and gestational age were found to be the most important predictors of a low 5-minute Apgar score [20].

The study population was subject to several forms of selection bias. First, our data have been collected from hospitals, which is why the prediction models are only relevant for institutional use. Poorer women, those of higher parity and those living far from a hospital are underrepresented among those who give birth in hospitals [41]. This may limit the generalisability of the models to hospital-based childbirths, as the study population may not fully represent all births, particularly those occurring outside health facilities. Second, the proportion of referred women was relatively high in some of the included hospitals, indicating a greater likelihood of complications and unfavourable birth outcomes within the dataset. 20.8% of parous women in the study population had a previous caesarean section, which may not be representative for wider populations. As women with a prior caesarean are more likely to deliver in facilities and be referred for institutional care, this proportion may reflect care-seeking and referral patterns different from the distribution in the wider pregnant population. As a result, the generalisability of the prediction models to the wider population of pregnant women in the region may be limited. Given the observed heterogeneity in baseline risk across countries, recalibration of the model intercept would be required prior to implementation in new settings to ensure accurate risk estimation. Overall, external validation in independent populations is needed to better understand the generalisability and transportability of the models.

In this study, a knowledge-based approach was used to select model predictors, aiming to enhance relevance for clinical decision-making. This strategy is known to improve the clinical applicability of the models by prioritising variables known to be clinically meaningful [40]. A limitation of this study is the potential for residual confounding due to unmeasured or imperfectly measured variables, as well as possible misclassification of maternal comorbidities, which may be underdiagnosed or inconsistently recorded given that screening for non-communicable diseases in many sub-Saharan African settings is often ad hoc and may occur only after symptom onset. Importantly, this study was designed for prediction rather than causal inference. Therefore, associations identified by the model should not be interpreted as causal relationships. Recent evidence has underscored the importance of accounting for country-level effects, given the observed variations in Apgar scores across settings [42]. Accordingly, country was included as a fixed effect to adjust for national-level differences. However, this adjustment did not account for residual heterogeneity at the hospital level. Random effects were not included, as they did not improve model performance.

There are several limitations related to the outcome being studied. The 5-minute Apgar score is an observational assessment, which is why it is inherently subjective and prone to inter-observer variation, although a standardised scale is used [43]. A limitation is the potential variability in Apgar scoring practices across hospitals and countries, which may introduce measurement error and outcome misclassification, potentially attenuating associations and affecting the model’s performance and comparability across settings. Furthermore, results of previous studies indicate differences in risk factors between low (≤ 3) and moderate (4–6) Apgar groups [44] and that Apgar scores 7–9 compared to 10 are associated with higher morbidity and mortality [20]. However, the use of a clinically meaningful cut-off aligns with common practice in medical research and reflects what is most relevant to the study setting. The Apgar score remains one of the most widely used and accessible indicators of neonatal condition in both clinical practise and research, particularly in low-resource settings where more sophisticated measurement methods may not be available. Its simplicity, rapid applicability, and long-standing clinical familiarity contribute to making it a valuable outcome measure.

A significant proportion of births with a low 5-minute Apgar score were stillbirths, nearly half of which were macerated stillbirths. This represents an important limitation but reflects the clinical reality of the study setting. Due to the limited availability of antenatal ultrasound, foetal viability prior to delivery cannot be reliably ascertained, making it difficult to distinguish between antepartum stillbirths and severely compromised live births at delivery. Excluding stillbirths would therefore risk misclassification, thereby reducing the clinical relevance of the models. The aim of this study was to predict low 5-minute Apgar scores using information available at admission, at a time when all included pregnancies still had the potential to result in a live birth. Excluding stillbirths based on postnatal data would therefore be inconsistent with the intended use of the model. This way, the models better reflect real-world conditions where this diagnostic uncertainty is uncommon, although this needs to be considered when interpreting the results.

The attenuation in model performance after excluding pregnancies with negative foetal heartbeat on admission suggests that the models capture both the risk of stillbirth and serious problems in newborns, not just complications among babies born alive. Accordingly, in low-resource settings where foetal viability cannot be reliably determined at admission, this broader predictive scope may be more clinically relevant for identifying high-risk pregnancies requiring urgent care. In addition, the ALERT data were not originally collected for predictive modelling purposes, which restricts the ability to exclude cases based on information only available after birth. Consequently, certain factors that might influence model performance in real-world applications could not be accounted for prospectively. Internal–external cross-validation showed that the parous model maintains acceptable discrimination across countries, with largely transportable predictor effects but some variation in baseline risk. The observed heterogeneity in performance across countries indicates that model accuracy may vary between settings.

### Interpretation

This study adds to the growing body of evidence supporting context-specific risk prediction tools tailored to low-resource settings. Existing prediction models for 5-minute Apgar scores were often tailored to specific subgroups of women, such as those with preterm [19] or very preterm [42] births. In contrast, this study focused on women with apparently normal pregnancies, as risk assessment is often most difficult in this group [21]. The decision curve analysis demonstrates that both models maintain a positive net benefit at lower threshold probabilities, suggesting it can effectively guide clinical interventions while minimising the resource burden associated with universal over-triage. A well-performing prediction model (AUC = 0.78) for 5-minute Apgar scores specifically to predict outcomes after induction of labour in vaginal deliveries was previously developed by Tarimo et al. for a low-resource setting in Tanzania. The most important predictors of a low 5-minute Apgar score in this population were maternal age, gestational age and birth weight [20]. Previous studies have described other possible risk factors for a low 5-minute Apgar score, some of which were not included as variables in the ALERT perinatal e-registry. Inclusion of foetal presentation and birthweight in a sensitivity analysis resulted in modest but consistent improvements in discrimination. The variables breech and transverse presentation were strong predictors, consistent with findings from previous models [7, 10, 44]. These findings suggest that model performance may be enhanced in settings where routine ultrasound information is available. Internal–external cross-validation indicated that adjustment of baseline risk may improve accuracy when applied in new settings.

## Conclusions

This study successfully developed two prediction models for births with low 5-minute Apgar scores in sub-Saharan Africa, one for nulliparous and one for parous women. Model performance was moderate but confirmed the feasibility of early detection of low Apgar score births based on admission data. These results also highlight the potential benefits of risk assessment by parity in maternity units. Overall, the results add to the growing body of evidence that simple, easily accessible tools can predict neonatal outcomes in low-resource settings. Future research should prioritise the external validation of these models within the sub-Saharan African setting. In addition, robust implementation studies are warranted to assess their context-specific applicability and impact. Well-performing prediction models represent only a first step toward more targeted and effective care, while intervention research needs to define the resulting surveillance and intervention packages addressing the higher risk. Interventions aimed at strengthening maternity units in sub-Saharan Africa, including improved triage routines and increased access to diagnostic tools such as routine ultrasound, could reduce both the incidence and implications of low Apgar scores in these regions.

## Supplementary Information

Supplementary Material 1.

## Data Availability

The perinatal e-registry data will become publicly accessible three years after the finalisation of the trial—thus, as of 31 December 2027—in accordance with the data management plan and publication policy. Prior to this date, data access may be granted upon reasonable request. Individuals seeking early access must submit a request to the ALERT steering committee. Access may be restricted if ALERT members are conducting or planning a similar analysis. The steering committee will review requests and respond within one month. If approved, the data will be released without delay.

## Abbreviations

ALERT: Action Leveraging Evidence to Reduce Perinatal Mortality and Morbidity in sub-Saharan Africa
CI: Confidence Interval
CITL: Calibration-in-the-Large
C-statistic: Concordance statistic
E:O: Expected-to-Observed ratio
HIV: Human Immunodeficiency Virus
LASSO: Least Absolute Shrinkage and Selection Operator
