# Supplementary material for: Prediction of low 5-minute Apgar scores: development and internal validation of parity-stratified clinical prediction models for sub-Saharan Africa
Source: BMC Pregnancy Childbirth. 2026 Apr 25;26:460. doi: 10.1186/s12884-026-09153-7 (PMC13110426; doi:10.1186/s12884-026-09153-7)
Supplement: Supplementary file 1 — Supplementary Material 1. [file 12884_2026_9153_MOESM1_ESM.docx]

Supplementary Table 1. Predictors included in final prediction models for low 5-min Apgar scores.

| Predictor | Definition | Coding |
| --- | --- | --- |
| Antenatal care visits | *Total number of antenatal visits made (regardless of the place of such visits) before admission for delivery.* | Continuous (number of visits) |
| Antepartum haemorrhage | *Vaginal bleeding at 28 weeks of gestation or later that is unrelated to labour and delivery.* | Yes / No |
| Cardiac/renal disease | *As marked on ANC card.* | Yes / No |
| Diabetes mellitus or  gestational diabetes | *Diabetes mellitus and/or gestational diabetes, as marked on ANC card* | Yes / No |
| Gestational age | *Best obstetric estimate of gestational age (in completed weeks) at delivery, based on an estimation from existing obstetrical data (preferred) or estimated by physical examination.* | Continuous (weeks) |
| HIV | *Status of mother at admission for delivery.* | Positive / Negative / Unknown |
| Hypertensive disorders | *As marked on ANC card.* | Yes / No |
| Last pregnancy outcome* | *Outcome of immediate previous pregnancy* | Abortion or miscarriage / Stillbirth /  Neonatal death / Baby alive at 28 days |
| Malaria | *As marked on ANC card.* | Yes / No |
| Maternal age | *Age of mother in completed years, recorded from hospital records or calculated from date of birth* | Continuous (years) |
| Parity* | *Number of previous deliveries (stillbirths and live births) excluding current delivery. Including both vaginal and c-section deliveries, excluding abortions or miscarriages.* | Continuous (number of births) |
| Pre-labour membrane rupture | *As marked on ANC card.* | Yes / No |
| Previous C-section* | *Previous caesarean section* | Yes / No |
| Severe anaemia | *Hb <7g/l. As marked on ANC card.* | Yes / No |
| Syphilis | *Status of mother at admission for delivery.* | Positive / Negative / Unknown |

ANC = Antenatal care. *Predictor only considered for the parous model.

Supplementary Table 2. TRIPOD Checklist: Prediction Model Development and Validation

| **Section/Topic** | **Item** |  | **Checklist Item** | **Page** |
| --- | --- | --- | --- | --- |
| **Title and abstract** | | | | |
| Title | 1 | D;V | Identify the study as developing and/or validating a multivariable prediction model, the target population, and the outcome to be predicted. | Section 1, Title page |
| Abstract | 2 | D;V | Provide a summary of objectives, study design, setting, participants, sample size, predictors, outcome, statistical analysis, results, and conclusions. | Section 2, Abstract |
| **Introduction** | | | | |
| Background and objectives | 3a | D;V | Explain the medical context (including whether diagnostic or prognostic) and rationale for developing or validating the multivariable prediction model, including references to existing models. | Section 3, Introduction |
|  | 3b | D;V | Specify the objectives, including whether the study describes the development or validation of the model or both. | Section 3, Introduction |
| **Methods** | | | | |
| Source of data | 4a | D;V | Describe the study design or source of data (e.g., randomized trial, cohort, or registry data), separately for the development and validation data sets, if applicable. | Section 4, paragraph 1 |
|  | 4b | D;V | Specify the key study dates, including start of accrual; end of accrual; and, if applicable, end of follow-up. | Section 4, paragraph 1 |
| Participants | 5a | D;V | Specify key elements of the study setting (e.g., primary care, secondary care, general population) including number and location of centres. | Section 4, paragraph 1 |
|  | 5b | D;V | Describe eligibility criteria for participants. | Section 4, paragraph 2 |
|  | 5c | D;V | Give details of treatments received, if relevant. | N/A |
| Outcome | 6a | D;V | Clearly define the outcome that is predicted by the prediction model, including how and when assessed. | Section 4, paragraph 3 |
|  | 6b | D;V | Report any actions to blind assessment of the outcome to be predicted. | N/A |
| Predictors | 7a | D;V | Clearly define all predictors used in developing or validating the multivariable prediction model, including how and when they were measured. | Supplementary materials |
|  | 7b | D;V | Report any actions to blind assessment of predictors for the outcome and other predictors. | N/A |
| Sample size | 8 | D;V | Explain how the study size was arrived at. | Section 4, paragraph 2 |
| Missing data | 9 | D;V | Describe how missing data were handled (e.g., complete-case analysis, single imputation, multiple imputation) with details of any imputation method. | Section 4, paragraph 5 |
| Statistical analysis methods | 10a | D | Describe how predictors were handled in the analyses. | Section 4, paragraph 4 |
|  | 10b | D | Specify type of model, all model-building procedures (including any predictor selection), and method for internal validation. | Section 4, paragraph 5 |
|  | 10c | V | For validation, describe how the predictions were calculated. | Supplementary materials |
|  | 10d | D;V | Specify all measures used to assess model performance and, if relevant, to compare multiple models. | Section 4, paragraph 6 |
|  | 10e | V | Describe any model updating (e.g., recalibration) arising from the validation, if done. | Section 4, paragraph 5 |
| Risk groups | 11 | D;V | Provide details on how risk groups were created, if done. | N/A |
| Development vs. validation | 12 | V | For validation, identify any differences from the development data in setting, eligibility criteria, outcome, and predictors. | Section 4, paragraph 6 |
| **Results** | | | | |
| Participants | 13a | D;V | Describe the flow of participants through the study, including the number of participants with and without the outcome and, if applicable, a summary of the follow-up time. A diagram may be helpful. | Section 4, paragraph 2  Section 5, paragraph 1 |
|  | 13b | D;V | Describe the characteristics of the participants (basic demographics, clinical features, available predictors), including the number of participants with missing data for predictors and outcome. | Section 5, paragraph 1 |
|  | 13c | V | For validation, show a comparison with the development data of the distribution of important variables (demographics, predictors and outcome). | Supplementary materials |
| Model development | 14a | D | Specify the number of participants and outcome events in each analysis. | Section 5, paragraph 1 |
|  | 14b | D | If done, report the unadjusted association between each candidate predictor and outcome. | N/A |
| Model specification | 15a | D | Present the full prediction model to allow predictions for individuals (i.e., all regression coefficients, and model intercept or baseline survival at a given time point). | Supplementary materials |
|  | 15b | D | Explain how to the use the prediction model. | Supplementary materials |
| Model performance | 16 | D;V | Report performance measures (with CIs) for the prediction model. | Section 5, paragraphs 2-3 |
| Model-updating | 17 | V | If done, report the results from any model updating (i.e., model specification, model performance). | Supplementary materials |
| **Discussion** | | | | |
| Limitations | 18 | D;V | Discuss any limitations of the study (such as nonrepresentative sample, few events per predictor, missing data). | Section 6, paragraphs 2-6 |
| Interpretation | 19a | V | For validation, discuss the results with reference to performance in the development data, and any other validation data. | N/A |
|  | 19b | D;V | Give an overall interpretation of the results, considering objectives, limitations, results from similar studies, and other relevant evidence. | Section 6, paragraph 7 |
| Implications | 20 | D;V | Discuss the potential clinical use of the model and implications for future research. | Section 6, paragraph 7 |
| **Other information** | | | | |
| Supplementary information | 21 | D;V | Provide information about the availability of supplementary resources, such as study protocol, Web calculator, and data sets. | Section 4, paragraph 1 |
| Funding | 22 | D;V | Give the source of funding and the role of the funders for the present study. | Section 1, Title page  Section 2, Abstract |

Supplementary Figure 1. Workflow for model development and validation.

Supplementary Table 3. Full model specification for prediction of 5-minute Apgar score in nulliparous women.

| **Variable** | | **Definition** | **Coding** | **Reference** | **Optimism-adjusted**  **Coefficient* (β)** | **(95% Confidence Interval)** | |
| --- | --- | --- | --- | --- | --- | --- | --- |
| Maternal age | | Age of mother in completed years, recorded from hospital records or calculated from date of birth | Continuous (years) |  | −0.014 | (−0.025, | −0.003) |
| Gestational age | | Best obstetric estimate of gestational age (in completed weeks) at delivery, based on an estimation from existing obstetrical data (preferred) or estimated by physical examination. | Continuous (weeks) |  | −0.127 | (−0.142, | −0.111) |
| Antenatal care visits | | Total number of antenatal visits made (regardless of the place of such visits) before admission for delivery. | Continuous (number of visits) |  | −0.049 | (−0.072, | −0.026) |
| Antepartum haemorrhage | | Vaginal bleeding at 28 weeks of gestation or later that is unrelated to labour and delivery. | Yes / No | No | 1.685 | (1.409, | 1.961) |
| Pre-labour membrane rupture | | As marked on ANC card. | Yes / No | No | −0.084 | (−0.298, | 0.129) |
| Hypertensive disorders | | As marked on ANC card. | Yes / No | No | 0.301 | (0.171, | 0.431) |
| Cardiac/renal diseases | | As marked on ANC card. | Yes / No | No | −0.054 | (−0.879, | 0.770) |
| Diabetes mellitus or gestational diabetes | | Diabetes mellitus and/or gestational diabetes, as marked on ANC card | Yes / No | No | −0.701 | (−1.382, | −0.020) |
| Syphilis | | Status of mother at admission for delivery. | Positive / Negative / Unknown | Negative |  |  |  |
|  | Positive |  |  |  | 0.516 | (−0.016, | 1.048) |
|  | Unknown |  |  |  | 0.221 | (0.134, | 0.307) |
| HIV | | Status of mother at admission for delivery. | Positive / Negative / Unknown | Negative |  |  |  |
|  | Positive |  |  |  | −0.136 | (−0.490, | 0.219) |
|  | Unknown |  |  |  | 0.204 | (0.081, | 0.326) |
| Malaria | | As marked on ANC card. | Yes / No | No | 0.280 | (0.006, | 0.555) |
| Severe anaemia | | Hb <7g/l. As marked on ANC card. | Yes / No | No | 0.848 | (0.545, | 1.152) |
| Country | |  | Benin / Malawi / Tanzania / Uganda | Tanzania |  |  |  |
|  | Benin |  |  |  | 1.278 | (1.112, | 1.444) |
|  | Malawi |  |  |  | 0.352 | (0.192, | 0.512) |
|  | Uganda |  |  |  | 0.968 | (0.805, | 1.132) |
| Model intercept (β_0)_ | |  |  |  | 1.659 |  |  |

*Coefficients are optimism-adjusted using bootstrap shrinkage.

Supplementary Table 4. Full model specification for prediction of 5-minute Apgar score in parous women.

| Variable | | Definition | Coding | Reference | Optimism-adjusted  Coefficient* (β) | (95% Confidence Interval) | |
| --- | --- | --- | --- | --- | --- | --- | --- |
| Maternal age | | Age of mother in completed years, recorded from hospital records or calculated from date of birth | Continuous (years) |  | −0.010 | (−0.017, | −0.003) |
| Gestational age | | Best obstetric estimate of gestational age (in completed weeks) at delivery, based on an estimation from existing obstetrical data (preferred) or estimated by physical examination. | Continuous (weeks) |  | −0.195 | (−0.207, | −0.182) |
| Antenatal care visits | | Total number of antenatal visits made (regardless of the place of such visits) before admission for delivery. | Continuous (number of visits) |  | −0.115 | (−0.135, | −0.096) |
| Parity | | Number of previous deliveries (stillbirths and live births) excluding current delivery. Including both vaginal and c-section deliveries, excluding abortions or miscarriages. | Continuous (number of births) |  | 0.120 | (0.096, | 0.144) |
| Antepartum haemorrhage | | Vaginal bleeding at 28 weeks of gestation or later that is unrelated to labour and delivery. | Yes / No | No | 1.981 | (1.830, | 2.133) |
| Pre-labour membrane rupture | | As marked on ANC card. | Yes / No | No | −0.154 | (−0.314, | 0.006) |
| Hypertensive disorders | | As marked on ANC card. | Yes / No | No | 0.431 | (0.324, | 0.538) |
| Cardiac/renal diseases | | As marked on ANC card. | Yes / No | No | 0.036 | (−0.484, | 0.557) |
| Diabetes mellitus or gestational diabetes | | Diabetes mellitus and/or gestational diabetes, as marked on ANC card | Yes / No | No | 0.002 | (−0.386, | 0.390) |
| Syphilis | | Status of mother at admission for delivery. | Positive / Negative / Unknown | Negative |  |  |  |
|  | Positive |  |  |  | 0.573 | (0.191, | 0.955) |
|  | Unknown |  |  |  | 0.140 | (0.066, | 0.214) |
| HIV | | Status of mother at admission for delivery. | Positive / Negative / Unknown | Negative |  |  |  |
|  | Positive |  |  |  | −0.277 | (−0.470, | −0.084) |
|  | Unknown |  |  |  | 0.237 | (0.133, | 0.341) |
| Malaria | | As marked on ANC card. | Yes / No | No | 0.050 | (−0.250, | 0.349) |
| Severe anaemia | | Hb <7g/l. As marked on ANC card. | Yes / No | No | 0.902 | (0.675, | 1.129) |
| Previous caesarean section | | Previous caesarean section*.* | Yes / No | No | −0.442 | (−0.525, | −0.359) |
| Last pregnancy outcome | | Outcome of immediate previous pregnancy | Abortion or miscarriage / Stillbirth / Neonatal death / Baby alive at 28 days | Baby alive at 28 days |  |  |  |
|  | Abortion/miscarriage |  |  |  | 0.146 | (0.033, | 0.260) |
|  | Stillbirth |  |  |  | 0.502 | (0.314, | 0.691) |
|  | Neonatal death |  |  |  | 0.276 | (0.043, | 0.508) |
| Country | |  | Benin / Malawi / Tanzania / Uganda | Tanzania |  |  |  |
|  | Benin |  |  |  | 1.276 | (1.144, | 1.408) |
|  | Malawi |  |  |  | 0.127 | (−0.011, | 0.266) |
|  | Uganda |  |  |  | 0.805 | (0.670, | 0.941) |
| Model intercept (β_0)_ |  |  |  |  | 4.305 |  |  |

*Coefficients are optimism-adjusted using bootstrap shrinkage.

Supplementary Table 5. Country- and hospital-specific characteristics of 124,376 mothers giving birth at 16 hospitals in Benin, Malawi, Tanzania and Uganda between July 2021 and December 2023.

| Country | Benin | | | | | Malawi | | | | | Tanzania | | | | | Uganda | | | | |
| --- | --- | --- | --- | --- | --- | --- | --- | --- | --- | --- | --- | --- | --- | --- | --- | --- | --- | --- | --- | --- |
| % |  | | | | 19.4 |  | | | | 36.1 |  | | | | 16.8 |  | | | | 27.7 |
| Hospital | 1 | 2 | 3 | 4 |  | 5 | 6 | 7 | 8 |  | 9 | 10 | 11 | 12 |  | 13 | 14 | 15 | 16 |  |
| n | 4481 | 12211 | 4460 | 3006 |  | 14444 | 13098 | 9434 | 7828 |  | 3240 | 8745 | 4949 | 4000 |  | 11727 | 2039 | 14856 | 5858 |  |
| % | 3.6 | 9.8 | 3.6 | 2.4 |  | 11.6 | 10.5 | 7.6 | 6.3 |  | 2.6 | 7.0 | 4.0 | 3.2 |  | 9.4 | 1.6 | 11.9 | 4.7 |  |
| Characteristics |  |  |  |  |  |  |  |  |  |  |  |  |  |  |  |  |  |  |  |  |
| Low 5-minute Apgar score | 9.0 | 14.3 | 10.8 | 3.9 | 11.4 | 5.1 | 3.0 | 4.8 | 4.2 | 4.3 | 4.8 | 2.6 | 1.5 | 1.4 | 2.4 | 8.5 | 14.4 | 7.5 | 3.9 | 7.6 |
| Maternal age  (years) | 26  (22-31) | 25  (21-30) | 29  (24-33) | 28  (24-32) | 26  (22-31) | 22  (19-28) | 22  (19-28) | 21  (19-26) | 22  (19-28) | 22  (19-28) | 27  (22-33) | 24  (20-31) | 25  (20-31) | 25  (20-32) | 25  (20-32) | 25  (21-30) | 25  (21-30) | 24  (20-29) | 23  (20-28) | 24  (20-29) |
| Antenatal care visits | 5  (3-6) | 4  (3-5) | 5  (4-6) | 4  (3-6) | 5  (3-6) | 4  (3-5) | 4  (3-4) | 3  (2-4) | 3  (3-4) | 4  (3-5) | 5  (4-6) | 5  (4-6) | 5  (4-6) | 6  (5-7) | 5  (4-6) | 4 (3-5) | 4  (3-5) | 4  (3-5) | 3  (3-4) | 4  (3-5) |
| Gestational age  (weeks) | 39  (38-40) | 38  (37-40) | 38  (37-39) | 39  (37-40) | 39  (37-40) | 38  (37-39) | 38  (37-38) | 38  (37-38) | 38  (37-38) | 38  (37-38) | 39  (38-39) | 40  (38-40) | 39  (37-40) | 39  (38-40) | 39  (38-40) | 39  (38-40) | 38  (38-39) | 38  (38-39) | 39  (38-40) | 38  (38-40) |
| HIV positive | 1.9 | 0.7 | 2.4 | 1.0 | 1.3 | 3.6 | 4.8 | 1.8 | 2.1 | 3.3 | 2.7 | 3.6 | 2.9 | 1.8 | 3.0 | 3.9 | 3.9 | 3.0 | 2.1 | 3.2 |
| Severe anaemia | 1.0 | 1.3 | 6.3 | 0.8 | 2.1 | 0.1 | 0.1 | 0.0 | 0.3 | 0.1 | 1.3 | 0.6 | 0.1 | 0.2 | 0.5 | 0.4 | 1.1 | 0.4 | 0.4 | 0.4 |
| Referral | 62.8 | 63.9 | 36.4 | 30.1 | 54.4 | 32.1 | 5.1 | 31.2 | 20.5 | 22.0 | 19.5 | 1.4 | 5.6 | 3.5 | 5.6 | 11.9 | 44.1 | 20.3 | 13.7 | 17.7 |
| Antepartum haemorrhage | 1.1 | 1.5 | 4.9 | 0.6 | 1.9 | 0.4 | 0.3 | 0.1 | 0.8 | 0.4 | 1.5 | 0.4 | 0.3 | 0.3 | 0.5 | 1.6 | 3.6 | 1.0 | 0.9 | 1.3 |
| Foetal heartbeat at admission | 95.4 | 91.0 | 93.8 | 93.3 | 92.6 | 91.0 | 98.3 | 96.2 | 97.1 | 95.3 | 96.3 | 98.7 | 97.2 | 99.0 | 98.0 | 93.5 | 81.7 | 97.4 | 95.7 | 94.8 |

Categorical variables are presented as percentages and continuous variables as medians with interquartile ranges (IQR)

Supplementary Figure 2a. Nomogram for estimating the probability of a low 5-minute Apgar score in nulliparous women, part 1.


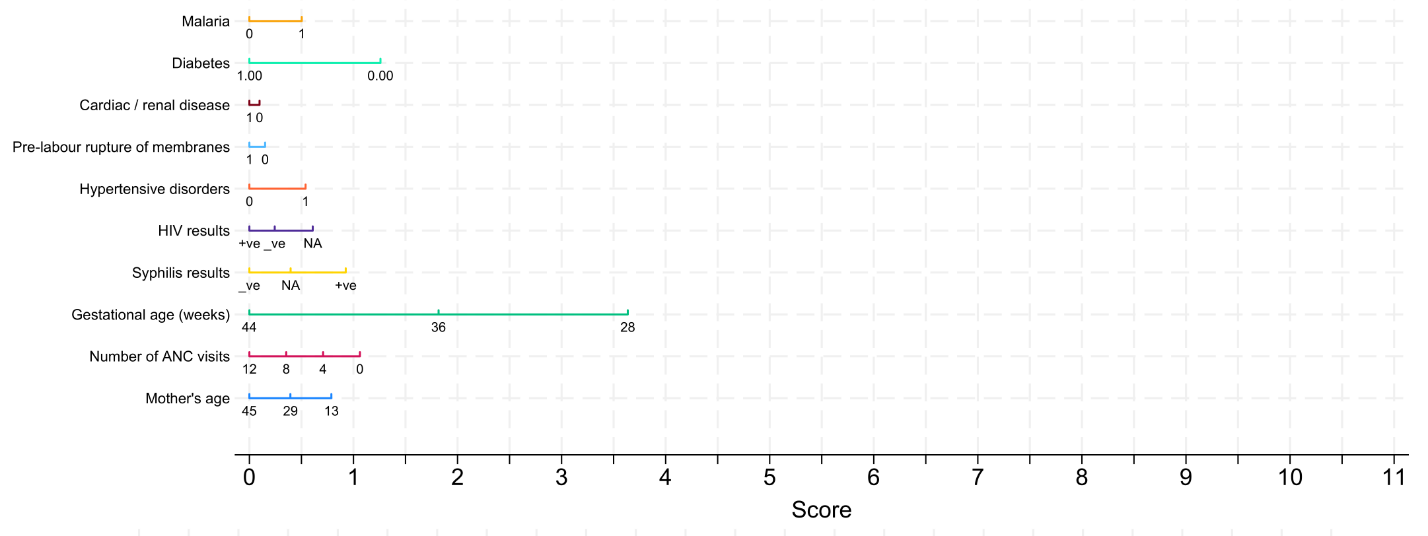


Supplementary Figure 2b. Nomogram for estimating the probability of a low 5-minute Apgar score in nulliparous women, part 2.


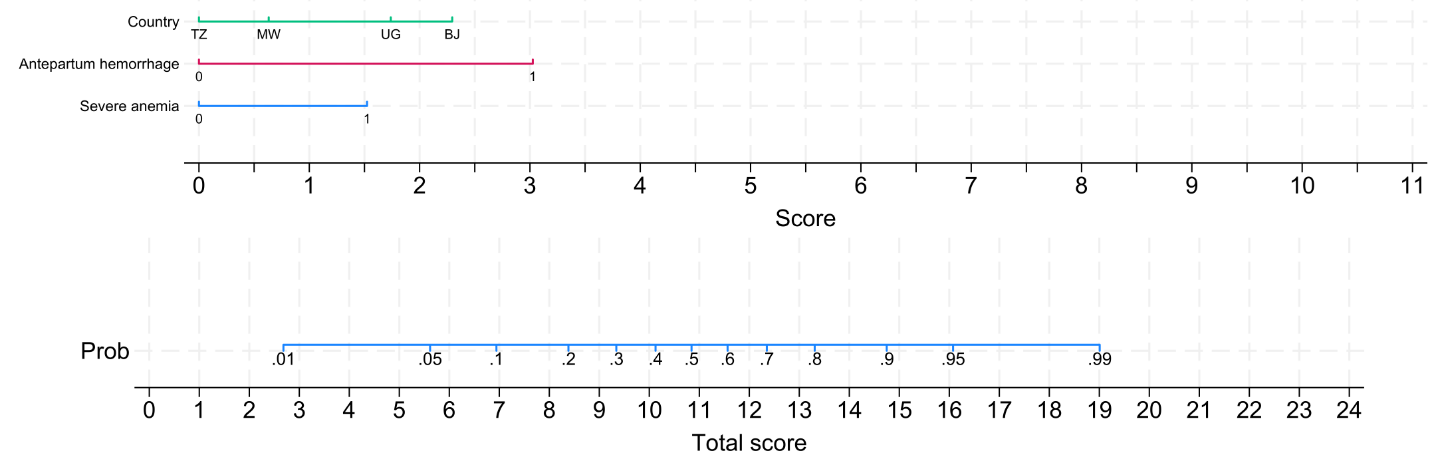


Supplementary Figure 3a. Nomogram for estimating the probability of a low 5-minute Apgar score in parous women, part 1.


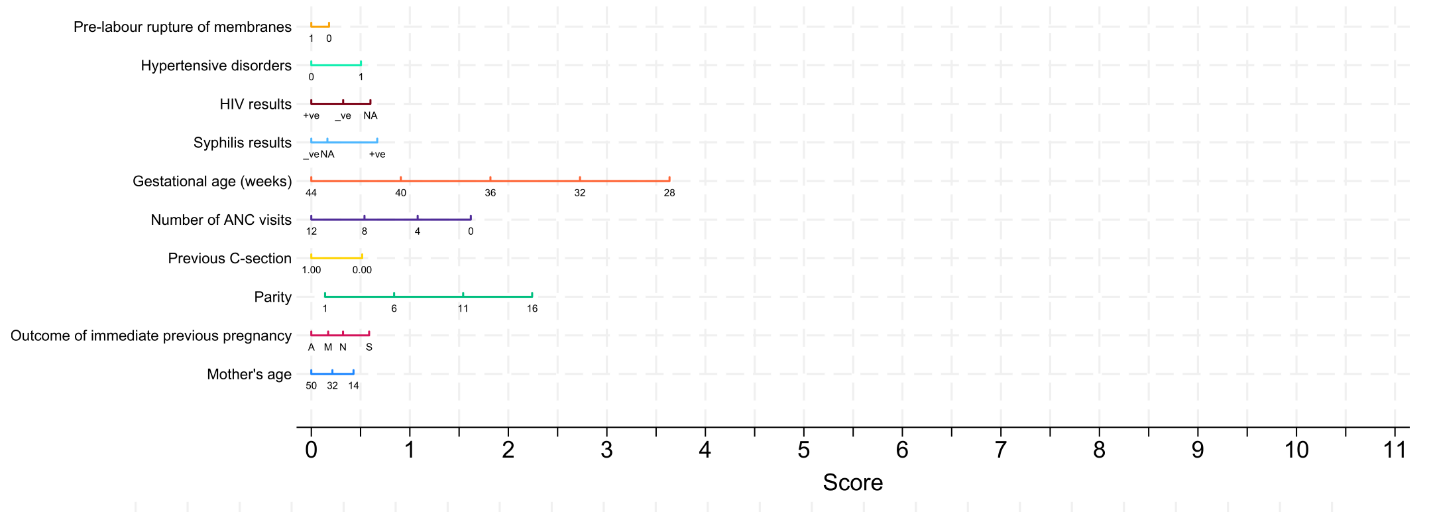


Supplementary Figure 3b. Nomogram for estimating the probability of a low 5-minute Apgar score in parous women, part 2.


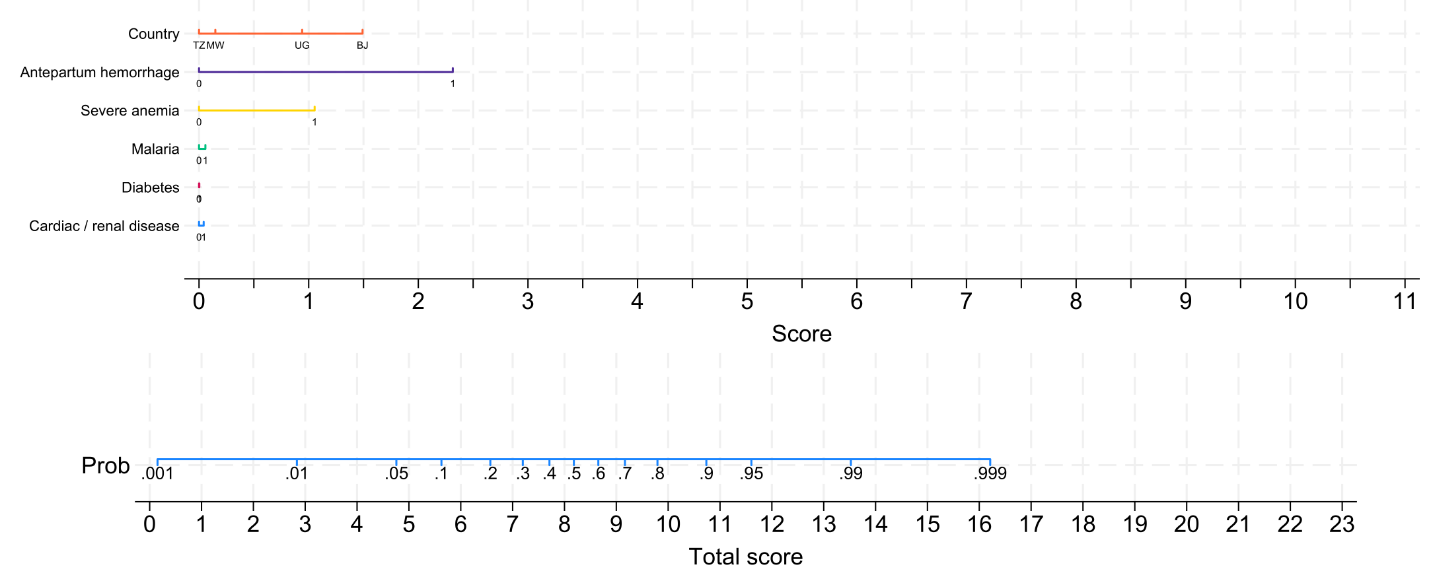


Supplementary Table 6. Associations between participant characteristics and missing predictor data.

|  | Odds ratio | (95% Confidence Interval) | | P-value* |
| --- | --- | --- | --- | --- |
| 5-minute Apgar score | 0.989 | (0.796, | 1.228) | 0.918 |
| Antenatal care visits | 0.967 | (0.713, | 1.310) | 0.827 |
| Country | 0.434 | (0.212, | 0.887) | 0.022 |
| Gestational age | 1.128 | (0.866, | 1.469) | 0.371 |
| HIV | 0.852 | (0.208, | 3.489) | 0.824 |
| Hypertensive disorders | 1.493 | (0.317, | 7.025) | 0.612 |
| Last pregnancy outcome | 1.323 | (0.631, | 2.775) | 0.459 |
| Malaria | 11.098 | (1.410, | 87.368) | 0.022 |
| Maternal age | 0.969 | (0.850, | 1.105) | 0.635 |
| Parity | 1.179 | (0.761, | 1.828) | 0.461 |
| Pre-labour membrane rupture | 3.300 | (0.690, | 15.791) | 0.135 |
| Previous caesarean section | 1.077 | (0.223, | 5.196) | 0.926 |
| Referral | 1.874 | (0.560, | 6.273) | 0.308 |
| Syphilis | 1.543 | (0.480, | 4.957) | 0.467 |

Odds ratios (OR) and 95% confidence intervals (CI) were derived from a logistic regression model with missingness (“any missing”) as the outcome. *P-values <0.05 were considered statistically significant.

Supplementary Table 7. Comparison of model discrimination (AUROC) between country fixed-effects and hospital random-effects models.

|  | **Fixed effect model (by country)** | **Random effects model**  **(by hospital)** |
| --- | --- | --- |
| Nulliparous model | 0.662 | 0.587 |
| Parous model | 0.732 | 0.686 |

Supplementary Table 8. Comparison of model performance after optimism-adjustment for nulliparous and parous participants across the control group, intervention group, and the full study population.

|  | **C-statistic** | **E:O ratio** | **Calibration slope** | **Calibration-in-the-large** |
| --- | --- | --- | --- | --- |
| **Nulliparous model** |  |  |  |  |
| Full study population | 0.662 | 0.990 | 0.983 | 0.003 |
| Control group | 0.676 | 0.993 | 0.974 | -0.001 |
| Intervention group | 0.655 | 1.005 | 0.975 | 0.003 |
| **Parous model** |  |  |  |  |
| Full study population | 0.732 | 0.993 | 0.994 | -0.001 |
| Control group | 0.742 | 1.007 | 0.989 | 0.001 |
| Intervention group | 0.729 | 0.993 | 0.992 | 0.001 |

Supplementary Figure 4. Optimism-adjusted calibration plot for prediction model for low 5-minute Apgar scores in nulliparous women (n=50106) with additional predictors: birth weight and foetal presentation.

Performance measures: C-statistic = 0.675. E:O radio = 0.997. Calibration slope = 0.983. CITL = 0.001. Scaled Brier score = 4.8%.

Supplementary Figure 5. Optimism-adjusted calibration plot for prediction model for low 5-minute Apgar scores in parous women (n=74270) with additional predictors: birth weight and foetal presentation.

Performance measures: C-statistic = 0.754. E:O radio = 0.999. Calibration slope = 0.996. CITL = 0.001. Scaled Brier score = 12.3%.

Supplementary Table 9. Coefficients of nulliparous model with additional predictors: birth weight and foetal presentation before and after internal validation.

| Predictor | | | Coefficient (β) | Odds ratio | Optimism-adjusted  Coefficient (β) | (95% Confidence Interval) | |
| --- | --- | --- | --- | --- | --- | --- | --- |
| Maternal age | | | −0.012 | 0.988 | −0.012 | (−0.023, | −0.001) |
| Gestational age | | | −0.057 | 0.944 | −0.056 | (−0.075, | −0.038) |
| Antenatal care visits | | | −0.039 | 0.962 | −0.039 | (−0.062, | −0.015) |
| Antepartum haemorrhage | | | 1.634 | 5.125 | 1.606 | (1.329, | 1.884) |
| Pre-labour membrane rupture | | | −0.127 | 0.881 | −0.125 | (−0.339, | 0.090) |
| Hypertensive disorders | | | 0.267 | 1.306 | 0.263 | (0.132, | 0.394) |
| Cardiac/renal diseases | | | −0.147 | 0.863 | −0.144 | (−0.980, | 0.691) |
| Diabetes mellitus or gestational diabetes | | | −0.619 | 0.538 | −0.608 | (−1.286, | 0.070) |
| Syphilis Reference: Negative | | |  |  |  |  |  |
| Positive | | | 0.510 | 1.666 | 0.502 | (−0.032, | 1.036) |
| Unknown | | | 0.234 | 1.263 | 0.230 | (0.143, | 0.317) |
| HIV Reference: Negative | | |  |  |  |  |  |
| Positive |  | | −0.139 | 0.870 | −0.137 | (−0.490, | 0.217) |
| Unknown | | | 0.180 | 1.197 | 0.177 | (0.054, | 0.299) |
| Malaria | | | 0.248 | 1.282 | 0.244 | (-0.035, | 0.523) |
| Severe anaemia | | | 0.789 | 2.202 | 0.776 | (0.469, | 1.083) |
| Foetal presentation Reference: Negative | | |  |  |  |  |  |
| Breech | | | 0.758 | 2.134 | 0.745 | (0.569, | 0.921) |
| Transverse | | | 1.131 | 3.097 | 1.111 | (0.428, | 1.795) |
| Birth weight | | | -0.001 | 0.999 | -0.001 | (-0.001, | 0.001) |
| Country Reference: Tanzania | | |  |  |  |  |  |
| Benin | |  | 1.237 | 3.445 | 1.216 | (1.050, | 1.382) |
| Malawi | | | 0.397 | 1.487 | 0.390 | (0.229, | 0.550) |
| Uganda | | | 1.067 | 2.905 | 1.049 | (0.885, | 1.212) |
| Model intercept (β_0_) | | | 0.505 |  | 0.453 | (0.415, | 0.491) |

Supplementary Table 10. Coefficients of parous model with additional predictors: birth weight and foetal presentation. before and after internal validation.

| Predictor | | Coefficient (β) | Odds ratio | Optimism-adjusted  coefficient (β) | | (95% Confidence Interval) | |
| --- | --- | --- | --- | --- | --- | --- | --- |
|  | |  |  |  | |  | |
| Maternal age | | −0.010 | 0.990 | −0.010 | (−0.017, | | −0.003) |
| Gestational age | | −0.088 | 0.916 | −0.088 | (−0.103, | | −0.072) |
| Antenatal care visits | | −0.100 | 0.905 | −0.099 | (−0.120, | | −0.079) |
| Parity | | 0.126 | 1.134 | 0.125 | (0.101, | | 0.150) |
| Antepartum haemorrhage | | 1.878 | 6.541 | 1.871 | (1.714, | | 2.027) |
| Pre-labour membrane rupture | | −0.267 | 0.765 | −0.266 | (−0.431, | | −0.102) |
| Hypertensive disorders | | 0.344 | 1.411 | 0.343 | (0.233, | | 0.452) |
| Cardiac/renal diseases | | −0.037 | 0.964 | −0.037 | (−0.575, | | 0.501) |
| Diabetes mellitus or gestational diabetes | | 0.065 | 1.067 | 0.065 | (−0.336, | | 0.466) |
| Syphilis  Reference: Negative | |  |  |  |  | |  |
| Positive |  | 0.553 | 1.738 | 0.551 | (0.165, | | 0.936) |
| Unknown |  | 0.171 | 1.186 | 0.170 | (0.095, | | 0.245) |
| HIV  Reference: Negative | |  |  |  |  | |  |
| Positive |  | −0.320 | 0.726 | −0.319 | (−0.515, | | −0.122) |
| Unknown |  | 0.174 | 1.190 | 0.174 | (0.068, | | 0.280) |
| Malaria | | −0.003 | 0.997 | −0.003 | (−0.310, | | 0.304) |
| Severe anaemia | | 0.907 | 2.478 | 0.904 | (0.674, | | 1.134) |
| Previous caesarean section | | −0.456 | 0.634 | −0.454 | (−0.539, | | −0.370) |
| Last pregnancy outcome  Reference: Baby alive at 28 days | |  |  |  |  | |  |
| Abortion/miscarriage |  | 0.096 | 1.101 | 0.096 | (−0.020, | | 0.212) |
| Stillbirth |  | 0.443 | 1.557 | 0.441 | (0.248, | | 0.634) |
| Neonatal death |  | 0.214 | 1.239 | 0.213 | (−0.027, | | 0.454) |
| Foetal presentation  Reference: Negative | |  |  |  |  | |  |
| Breech | | 0.951 | 2.588 | 0.947 | (0.828, | | 1.066) |
| Transverse | | 1.009 | 2.743 | 1.005 | (0.661, | | 1.350) |
| Birth weight | | −0.001 | 0.999 | −0.001 | (−0.001, | | −0.001) |
| Country  Reference: Tanzania | |  |  |  |  | |  |
| Benin |  | 1.208 | 3.347 | 1.203 | (1.069, | | 1.337) |
| Malawi |  | 0.226 | 1.253 | 0.225 | (0.084, | | 0.365) |
| Uganda |  | 0.949 | 2.584 | 0.945 | (0.808, | | 1.083) |
| Model intercept (β_0_) |  | 2.317 |  | 2.299 | (2.267, | | 2.331) |

Supplementary Table 11. Internal-external-cross-validation performance in parous model

|  | **Discrimination (AUC)** | **Calibration-in-the-large** | **Calibration slope** |
| --- | --- | --- | --- |
| Benin | 0.69 | 1.18 | 0.88 |
| Malawi | 0.58 | 0.11 | 0.74 |
| Tanzania | 0.67 | −0.80 | 1.11 |
| Uganda | 0.68 | 0.77 | 0.97 |
| Pooled performance | 0.65 | 0.32 | 0.92 |
| Tau^2^ | 0.00 | 0.74 | 0.02 |
| I^2^ (%) | 96.50 | 99.85 | 92.67 |
